# Impact of Ocular Massage on Intraocular Pressure and Schlemm Canal Dimensions in Healthy Adults: Protocol for a Randomized Controlled Trial

**DOI:** 10.2196/78864

**Published:** 2026-02-26

**Authors:** Mingxuan Zhang, Luning Qin, Liangzhang Tan, Yongtao Li, Qing Zhang, Shuhan Wang, Zhihui Zhang, Emmanuel Eric Pazo, Xinjun Ren

**Affiliations:** 1 Department of Ocular Trauma Tianjin Key Laboratory of Retinal Functions and Diseases, Tianjin Branch of National Clinical Research Center for Ocular Disease, Eye Institute and School of Optometry, Tianjin Medical University Eye Hospital Tianjin China; 2 Tianjin Key Laboratory of Retinal Functions and Diseases, Tianjin Branch of National Clinical Research Center for Ocular Disease, Eye Institute and School of Optometry, Tianjin Medical University Eye Hospital Tianjin China; 3 Independent Consultant Shanghai China; 4 Department of Clinical Research Tianjin Key Laboratory of Retinal Functions and Diseases, Tianjin Branch of National Clinical Research Center for Ocular Disease, Eye Institute and School of Optometry, Tianjin Medical University Eye Hospital Tianjin China

**Keywords:** ocular massage, Schlemm canal, trabecular meshwork, intraocular pressure, aqueous humor

## Abstract

**Background:**

Ocular massage has been reported to lower intraocular pressure (IOP) temporarily. This effect could be related to enhanced aqueous humor outflow; however, the mechanism is unclear.

**Objective:**

This study aims to examine the impact of digital and EyePeace ocular massage on IOP fluctuations and investigate whether the observed reduction in IOP is attributable to morphological changes in the Schlemm canal (SC).

**Methods:**

Participants were randomly assigned in a 1:1:1 ratio to 1 of 3 groups: digital ocular massage, EyePeace ocular massage, or no ocular massage. The primary outcome measure will be IOP. The secondary outcome measures will include anterior segment optical coherence tomography assessment of the SC and trabecular meshwork. In addition, adverse events in the quality of vision will be monitored and documented using a mobile app–based questionnaire. All assessments will be performed at baseline and 10 minutes after the assigned interventions.

**Results:**

Data collection was completed in the summer of 2025. The study results are expected to be published by the end of 2025.

**Conclusions:**

This study will investigate the impact of ocular massage on IOP and SC dimensions by using a randomized controlled design. As preliminary evidence suggests that ocular massage may transiently reduce IOP by enhancing aqueous humor outflow, this study will aim to clarify the underlying mechanism. By comparing digital ocular massage, EyePeace ocular massage, and no ocular massage, the study will assess changes in IOP and SC morphology. The findings are expected to provide insights into the role of mechanical manipulation in modulating aqueous humor outflow, potentially informing nonpharmacological strategies for possible glaucoma management.

**Trial Registration:**

Chinese Clinical Trial Registry ChiCTR2400093512; https://www.chictr.org.cn/showproj.html?proj=250459

**International Registered Report Identifier (IRRID):**

DERR1-10.2196/78864

## Introduction

### Background

Ocular massage has been recognized as a potential nonpharmacological technique to transiently reduce intraocular pressure (IOP) across various clinical scenarios. It has notably been used after glaucoma surgeries such as trabeculectomy to manage postoperative pressure fluctuations [1,2] presumably through the expansion of filtration blebs, enhancing aqueous humor drainage [3,4]. Moreover, ocular massage has shown efficacy in managing acute elevations of IOP after intravitreal injections and in acute angle-closure scenarios, demonstrating its versatility in ophthalmic practice [5,6].

In the setting of acute angle-closure attacks, patients can be instructed to perform ocular massage as an initial intervention to transiently lower IOP before definitive medical or surgical treatment can be administered [7,8]. Despite these promising clinical applications, previous studies have limitations that hinder drawing definitive conclusions about the underlying mechanisms and the sustained effectiveness of ocular massage [6,7]. Although the prior studies demonstrated temporary reductions in IOP after digital ocular massage, they were limited by the wide variability in the pressure applied by clinicians or patients and in the duration and frequency of massage because even small variations in finger placement or pressure can result in unpredictable IOP responses. These findings collectively highlight the potential clinical applications of ocular massage in various ophthalmic contexts [9]. EyePeace (Eye Comfort Ltd) is a commercially available device for eyelid massage designed to enhance meibum expression for dry eye disease related to meibomian gland dysfunction [10,11]. This flexible, handheld silicone device is used in conjunction with warm compress therapy and applies controlled vertical pressure to the closed eyelids [10,11]. EyePeace addresses the key limitations of manual digital ocular massage by offering a standardized, safe, and reproducible alternative. Unlike digital ocular massage, which is limited by inconsistent finger pressure, variable technique, and anatomical inaccuracy, EyePeace delivers calibrated and uniform mechanical force to the eyelids, ensuring consistent application across users and sessions. Its ergonomic design promotes proper positioning and motion, improving the reliability of therapeutic outcomes. By operating within safe pressure thresholds, EyePeace minimizes the risk of ocular complications such as hypotony, optic nerve stress, or retinal damage that may result from manual overcompression. In addition, its ease of use facilitates home-based therapy, enhancing patient adherence and long-term management. Originally developed for meibomian gland dysfunction, the device may also support aqueous humor outflow through gentle scleral compression, suggesting potential utility in select glaucoma settings. Overall, EyePeace offers a more controlled and patient-friendly approach that overcomes the variability and safety concerns associated with digital ocular massage.

IOP homeostasis is primarily dictated by the dynamic balance between aqueous humor production and outflow. The conventional drainage pathway involves the trabecular meshwork (TM), which directs aqueous humor into the Schlemm canal (SC) and then into aqueous and episcleral veins via collector channels [1,5]. A secondary, unconventional uveoscleral outflow pathway also contributes to aqueous humor clearance, albeit to a lesser extent [2,3,11-13]. Previous studies have provided insights into anatomical variations in aqueous humor drainage structures and their implications for IOP regulation [4,14]. Kagemann et al [15] reported that the cross-sectional area of the SC was significantly reduced in patients with glaucoma compared to healthy individuals, suggesting a structural contribution to elevated IOP [6,7,16,17]. Similarly, Chung et al [18] found that patients with newly diagnosed open-angle glaucoma who had larger baseline SC areas exhibited a more substantial IOP-lowering response to topical hypotensive agents, whereas TM width did not seem to influence the degree of pressure reduction. Advances in high-resolution imaging have now made it possible to visualize the corneoscleral region, including the SC and TM [19,20].

### Objectives

This study aims to evaluate changes in IOP after the use of the EyePeace ocular massager. We hypothesize that EyePeace will achieve a more consistent and pronounced IOP reduction compared to digital ocular massage. Furthermore, the observed IOP reduction may be correlated with morphological changes in underlying ocular structures.

## Methods

### Study Design

This single-masked, randomized controlled trial will enroll 36 healthy Chinese participants (72 eyes) aged 18 years or older. They will be randomly assigned in a 1:1:1 ratio to the digital ocular massage group (24 eyes), EyePeace ocular massage group (24 eyes), or no ocular massage (24 eyes). The trial will be conducted at Tianjin Medical University Eye Hospital (TMUEH) in Tianjin, China, a nationally accredited clinical trial site. Healthy participants who voluntarily consent to undergo ocular massage will be invited to join this study to assess noninvasive clinical parameters. Patients and the public will not be involved in the design, implementation, reporting, or dissemination of this study. This study was registered on the Chinese Clinical Trial Registry (ChiCTR2400093512) on December 6, 2024.

### Eligibility Criteria

The inclusion criteria were as follows: (1) age 18 years or older; (2) willingness to provide written informed consent, accept random allocation, and participate in ocular massage interventions, follow-ups, and assessments; and (3) IOP within the target range of 10 to 21 mm Hg. Patients were excluded based on the following criteria: (1) prior diagnosis of an eye disease, (2) history of ocular surgery, (3) current use of medications that might affect anterior segment ocular anatomy, (4) current contact lens wear, (5) history of ocular allergy, and (6) systemic disorders [21,22].

### Interventions

Eligible participants will be randomly assigned in a 1:1:1 ratio to (1) digital ocular massage (group A), (2) EyePeace ocular massage (group B), or (3) no ocular massage (group C). Group A and group B participants will receive ocular massage for 10 minutes [5,10,11]. Any participant experiencing an adverse event (AE) will be withdrawn from the trial, documented, provided appropriate treatment, and referred immediately to the principal investigator (PI). Clinical research staff will receive professional training covering participant enrollment, treatment procedures (including risks and benefits), and data collection processes. Participants will be sufficiently informed about the study and provided with compliance education to enhance participation and minimize noncompliance. Study staff will proactively contact participants in advance by telephone or SMS text message. EyePeace is a flexible, medical-grade silicone device designed to apply gentle, pulsatile vertical pressure to the closed eyelids. Although it does not contain a built-in pressure gauge, manufacturer specifications suggest that the applied force typically ranges from 0.2 to 0.5 N. To ensure consistency, all participants in the EyePeace ocular massage group received standardized instructions and a demonstration before use. In addition, a trained examiner supervised the massage procedure to ensure correct technique and duration. For the digital ocular massage group, clinical staff applied consistent fingertip pressure guided by prior training and feedback from pilot sessions, aiming to replicate the mechanical effect of the EyePeace ocular massage device. Although exact biomechanical equivalence between digital ocular massage and EyePeace ocular massage cannot be assured, the standardization procedures were designed to ensure comparability in massage duration, eyelid area coverage, and application frequency.

### Provisions for Posttrial Care

Participants who experience symptoms of conjunctivitis, keratitis, or other adverse reactions during the study or within 6 months after its completion will receive timely, targeted treatment. All such events will be documented in the AE record.

### Primary Outcome: IOP

The study’s primary outcome is assessing IOP (mm Hg) before and after the intervention. All participants will undergo ophthalmologic examinations on the same day. A slit lamp examination will first be performed on all eyes, followed by IOP measurement in the sitting position, using an iCare IC100 rebound tonometer (iCare Finland Oy) [19]. The instrument will be calibrated regularly according to the manufacturer’s instructions. A disposable, single-use probe was loaded into the device and aligned 4 to 8 mm perpendicular to the central cornea. Six consecutive measurements were performed. The software automatically discarded the highest and lowest values, and the IOP was calculated from the remaining 4 values. Only proper measurements (indicated by a green background within acceptable limits) were included.

### Secondary Outcomes

#### Best Corrected Visual Acuity

Best corrected visual acuity, reported in logarithm of the minimum angle of resolution units, will be measured using a standardized visual acuity chart under controlled lighting conditions to assess any changes due to ocular massage [10,20]. Baseline and postintervention measurements will be recorded to determine the impact on visual function.

#### SC Area and SC Diameter

SC area (μm^2^) and SC diameter (μm) will be analyzed using anterior segment optical coherence tomography (AS-OCT) [11,23]. High-resolution images of the temporal corneoscleral region will be captured at baseline and after the intervention, ensuring consistency in scanning alignment. SC area will be delineated manually using ImageJ software, while SC diameter will be measured from the anterior to posterior endpoints of the canal [12,24].

#### TM Width and TM Thickness

TM parameters, including width (μm) and thickness (μm), will be assessed from AS-OCT images [13]. TM width will be defined as the distance between the scleral spur and the Schwalbe line, whereas TM thickness will be measured at the anterior endpoint and midpoint of the SC [14,25].

#### Mobile App–Based Quality of Vision Questionnaire

The original Quality of Vision Questionnaire includes 10 visual symptoms, measured in terms of frequency, severity, and impact on daily activities. It was designed to assess patients’ subjective visual experience, especially after refractive or cataract surgery [26]. In this study, participants will complete the Quality of Vision Questionnaire [26] before and after ocular massage to assess subjective visual disturbances, visual clarity, and visual discomfort. The questionnaire will be delivered via a web application on the WeChat platform (Tencent Holdings Ltd) [27]. An independent examiner will perform all measurements to minimize bias, and data will be analyzed for statistical significance using appropriate paired comparison tests. In addition to the Quality of Vision Questionnaire, 3 items will be administered to measure overall quality of vision score (Likert scale, 0-10), ocular pain score (Likert scale, 0-10), and ocular discomfort score (Likert scale, 0-10; Multimedia Appendix 1).

### Participant Timeline

**Figure 1:**
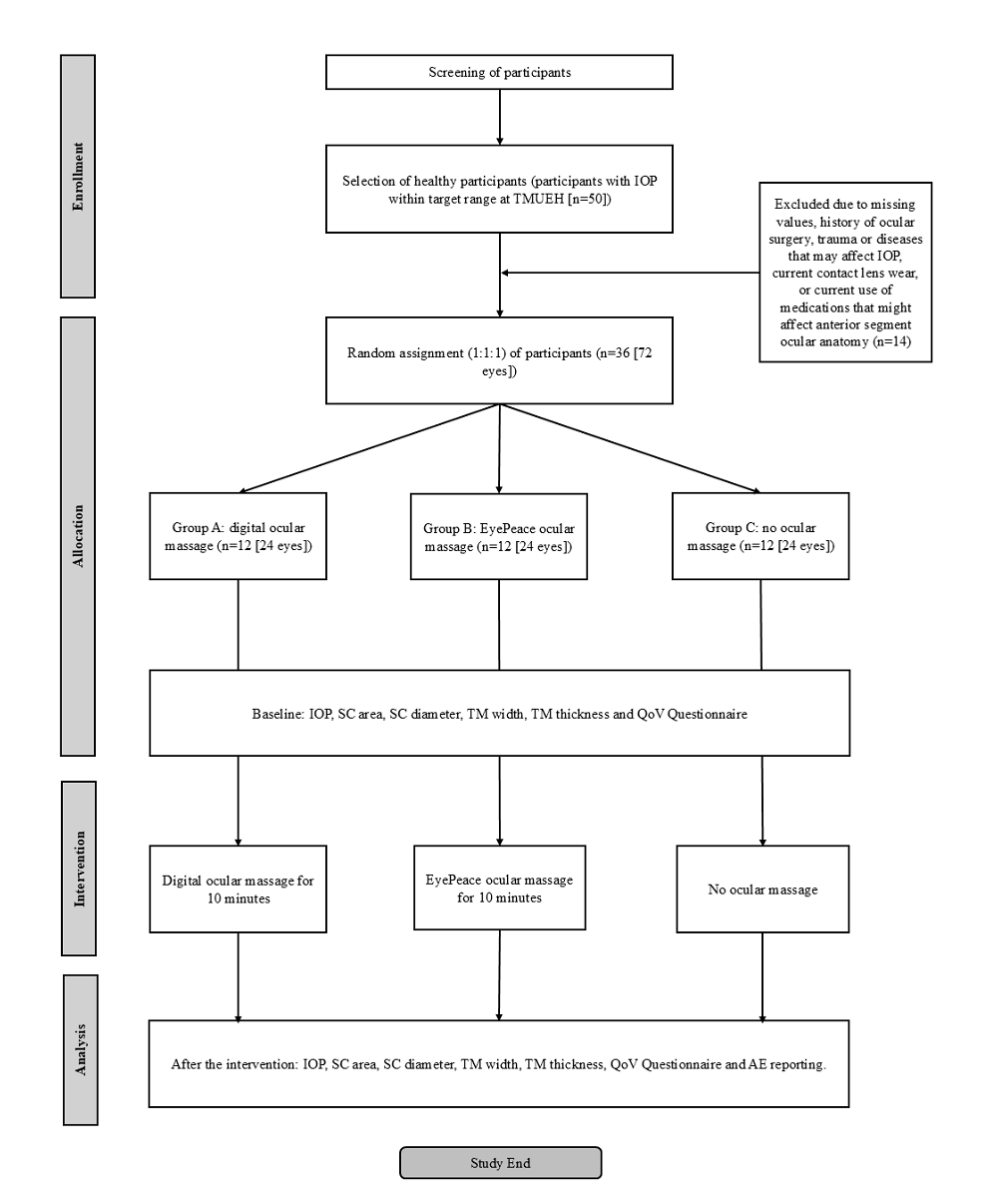
Study design flowchart. AE: adverse event; IOP: intraocular pressure; QoV: Quality of Vision; SC: Schlemm canal; TM: trabecular meshwork; TMUEH: Tianjin Medical University Eye Hospital.

During the study, participants undergo ocular examinations at baseline and 10 minutes after the intervention. The study procedures for participant recruitment, intervention, assessment, and data follow-up follow the SPIRIT (Standard Protocol Items: Recommendations for Interventional Trials) guidelines (Table 1) [28]. The study process is illustrated in Figure 1.

**Table 1:** Study procedures conducted in accordance with the SPIRIT (Standard Protocol Items: Recommendations for Interventional Trials) guidelines.

Time point			Study period						
			Enrollment		Allocation		After allocation		
			Recruitment (2-4 wk)		Random assignment (0 d)		Baseline		Follow-up
**Enrollment**									
	Eligibility screening	✓							
	Informed consent	✓							
	Random assignment			✓					
**Intervention**									
	Digital ocular massage group					✓		✓	
	EyePeace ocular massage group					✓		✓	
	No ocular massage group					✓		✓	
**Assessments**									
	Intraocular pressure (mm Hg)					✓		✓	
	Best corrected visual acuity (logarithm of the minimum angle of resolution)					✓		✓	
	Schlemm canal area (μm^2^)					✓		✓	
	Schlemm canal diameter (μm)					✓		✓	
	Trabecular meshwork width (μm)					✓		✓	
	Trabecular meshwork thickness (μm)					✓		✓	
	Quality of Vision Questionnaire score					✓		✓	

### Sample Size

On the basis of the stated assumptions (a common SD of 1.9 and a maximum mean difference in IOP between groups of approximately 2 mmHg), a total of 36 participants (72 eyes [participants will contribute both eyes unless exclusion criteria apply]; 3 groups; 24 eyes per group) are required to achieve 90% power at α=.05 [1,5]. This corresponds to 3 groups of equal size in a randomized controlled trial. The calculations use a 1-way analysis of variance to test overall mean differences and consider a moderate to large effect size in IOP reduction. Allowing for a 10% dropout rate, the recruitment target is 40 participants (80 eyes). If further data refine the expected mean difference, the sample size will be adjusted accordingly (larger samples for smaller expected differences and vice versa).

### Recruitment

This study will be conducted from March to June 2025 at TMUEH. Healthy participants in Tianjin, China, will be invited to participate. Recruitment will be carried out through posters, online advertisements, and social media platforms. Specialized professionals will collect demographic information of the participants during the initial screening.

### Allocation

The patients will be randomly assigned to 1 of 3 groups: digital ocular massage (group A), EyePeace ocular massage (group B), or no ocular massage (group C). Randomization will be performed by a statistician using a network-based randomization procedure with a fixed block size of 4 to ensure balanced group allocation. Group assignments will be placed in sealed, opaque envelopes held by the PI. Each envelope will include the participant’s information and intervention assignment.

This trial will use a single-masked design. Participants will be aware of their assigned intervention (digital ocular massage, EyePeace ocular massage, or no ocular massage). However, the data analysis team and clinical assessment staff will remain masked to group allocation. All clinical assessments will be performed by a masked examiner who is not involved in data collection or group allocation. The conditions and protocols for disclosure of group information, if required, will be established and overseen by the PI.

### Data Collection and Management

The clinical assessment staff will collect data on IOP (mm Hg), best corrected visual acuity (logarithm of the minimum angle of resolution), SC area (μm^2^), SC diameter (μm), TM width (μm), and TM thickness (μm), as well as responses to the Quality of Vision Questionnaire. Cases that are discontinued will be retained for subanalysis; these cases will be particularly useful for evaluating outcomes such as AEs and adherence. All participants will undergo a comprehensive ophthalmic examination at baseline and after the intervention. When participants enroll for the study, trained clinical researchers and outpatient physicians participating in the trial will enter participant information into electronic case report forms. All study data will be entered into a specialized management program and stored securely in a database to ensure confidentiality. Designated statisticians from the TMUEH research team will analyze the anonymized data according to the trial plan. Participants will be informed in advance and encouraged to actively cooperate with study procedures to minimize attrition. Although AEs are unlikely in this study, the intervention will be suspended upon detection of any ocular symptoms of concern during slit lamp examination. Treatment for the AE, along with regular telephone and SMS text message updates, will be provided to those affected. All AEs are documented and reported to the trial steering committee. Participants unable to complete subsequent follow-up will be withdrawn from the study. Those who experience intolerable side effects will receive treatment and be removed immediately from the study.

### Statistical Methods

Following the guidelines for statistical analysis plans in clinical trials [18,29], all statistical analyses will be conducted using SPSS software (version 25.0; IBM Corp). The significance level is set at 5% (2-tailed), with a 95% CI. The normality of data distribution will be assessed using the Shapiro-Wilk test. Data will be compared using 2-way repeated measures analysis of variance. If the hypothesis is unmet (*P*>.05), the Greenhouse-Geisser correction will be applied. Multiple comparisons will be made between groups at different time points using the Bonferroni-Holm correction. If the Shapiro-Wilk test indicates that the data are not normally distributed (*P*<.05), nonparametric tests will be used: paired comparisons will use the Wilcoxon signed-rank test, and between-group comparisons will use the Kruskal-Wallis H test. Statistical significance will be considered at *P*<.05 for 2-tailed tests unless otherwise specified. To ensure participant safety, the distribution of AEs will be described for both digital ocular massage and EyePeace ocular massage groups. The incidence rates of AEs were compared using the chi-square test or the Fisher exact test, as appropriate. An intention-to-treat analysis will be the primary analytic approach. All participants will be included in the analysis based on their original allocation, regardless of protocol adherence. For missing data, multiple imputation will be performed using the fully conditional specification method, assuming data are missing at random. A sensitivity analysis using a per-protocol approach will also be conducted to compare results. Given the short duration of this study, an interim analysis will not be performed.

### Ethical Considerations

This study will adhere to the ethical principles outlined in the Declaration of Helsinki and has been approved by the TMUEH Institutional Review Board (2024KY-56). Informed consent will be obtained by clinical physicians who have received specialized training. Written informed consent will be obtained from all participants before their participation. All information and clinical data, including participants’ personal medical histories, will remain confidential.

### Monitoring

The trial steering committee, comprising project leaders and key decision-makers, is responsible for overseeing and supporting the study. This committee will provide strategic guidance throughout all phases of the research and will be responsible for preparing and submitting the final study report.

Given the low likelihood of AEs in healthy participants, a data monitoring committee will not be established. Data collection and management will be conducted using a Microsoft Excel (version 16) database, which will be regularly updated in accordance with the study protocol.

Any modifications to the inclusion criteria or test procedures will be submitted to the TMUEH Medical Ethics Review Committee for review and approval before implementation.

### Classification of AEs

Any unexpected symptoms or signs that may pose a risk to the physical or mental well-being of participants will be classified as AEs. Localized AEs may include intolerable dry eye symptoms, keratitis, severe corneal ulcers, conjunctivitis, eye irritation, and blurred vision. Mild AEs, such as temporary dry eyes or foreign body sensation, which do not affect daily activities, will be managed by suspending the intervention, administering artificial tears, and re-examining participants after 30 minutes. Moderate AEs, such as conjunctivitis or slightly elevated IOP, will be managed by suspending the treatment, referring to the PI, providing targeted treatment, and following up until the symptoms resolve. In the event of a serious AE, the PI and the TMUEH Medical Ethics Review Committee will be promptly notified, the trial will be immediately suspended, and appropriate medical intervention will be provided to ensure participant safety and minimize further risk.

### Auditing Trial Conduct

This study will be subject to weekly evaluations and assessments conducted by independent supervisors to ensure compliance with the research protocol and ethical standards.

### Dissemination Plans

The research findings will be disseminated to relevant stakeholders, including participants, their families, physicians, advisory committees, and medical boards. In addition, the results will be shared through high-impact medical journals and presentations at international medical conferences to ensure broad academic and clinical engagement.

## Results

As of July 15, 2025, the study recruited 50 participants, and all provided written informed consent. The initial results are anticipated to be reported and published after the completion of data collection and analysis, with publication expected in 2025-2026.

## Discussion

### Summary

Eye massage is commonly used for relaxation, relief of eye strain, and the management of dry eye syndrome [19,30]. However, improper technique or excessive pressure can lead to ocular complications [20,31]. A case study by Yadgari et al [32] reported the formation of an intracorneal cleft after ocular massage in a patient who had undergone Ahmed valve implantation for glaucoma, highlighting the risk of corneal structural damage when excessive external pressure is applied to the eye. Riede-Pult et al [33] investigated the short-term effects of eyelid massage on corneal shape using topographic analysis. Although the study found no significant short-term changes in corneal curvature or visual acuity, it did not assess potential long-term consequences, suggesting that while brief eyelid massage may be safe, repeated or forceful manipulation could carry risks. A systematic review by Yin et al [34] explored the adverse effects of massage therapy in pain-related conditions. Reported complications included soft tissue trauma, nerve injury, and vascular complications, which could theoretically apply to forceful eye massage as well. The possibility of pressure-induced damage to the delicate ocular and periocular structures warrants caution. Lee et al [30] conducted a randomized controlled trial comparing a thermal eye massager with artificial tears to treat dry eye syndrome. While the thermal massage was found to be effective and generally safe, some patients experienced mild and transient adverse effects such as irritation or temporary discomfort. A broader review assessed the safety of massage therapy and highlighted rare but serious adverse effects such as vascular damage, nerve compression, and eye trauma [24,35]. While these events are uncommon, they emphasize the importance of appropriate technique and caution when applying pressure around the eyes [25,26,36,37]. This study will enroll only healthy adult participants with IOP within the target range of 10 to 21 mm Hg and no ocular comorbidities to minimize confounding factors and ensure a controlled baseline for evaluating the isolated effects of ocular massage on the SC and IOP. However, we acknowledge that this limits the generalizability of the findings, particularly to populations with glaucoma or postsurgical eyes, where ocular massage may be used therapeutically. Future studies in these clinical populations are warranted to validate the safety and efficacy of these interventions under real-world conditions. In this randomized controlled trial, participants were randomly assigned to 1 of 3 groups (digital ocular massage, EyePeace ocular massage, or no ocular massage) and underwent baseline and 10-minute postintervention assessments using AS-OCT and IOP measurements. These short-term findings could inform novel, nonpharmacological approaches for glaucoma risk mitigation. However, the short follow-up window limits conclusions regarding durability. Future work should incorporate longer follow-up periods, include patient populations with glaucoma or ocular hypertension, and explore the correlation between morphological changes and sustained IOP regulation. Safety assessments and comparative efficacy in real-world clinical settings remain crucial.

A more extended follow-up period is needed to determine the long-term effects of digital ocular massage and EyePeace ocular massage on IOP and anterior segment anatomical changes. It is also unclear whether the changes in IOP, anterior segment anatomy, and vision are sustained for long periods and can be considered long-term therapy. This study is a single-masked clinical trial; therefore, potential bias may still be present, despite the researcher’s objective evaluation of data. In addition, as this study measures outcomes only 10 minutes after the intervention, it may not capture the longer-term persistence or fluctuation of the IOP-lowering effect. Future studies with extended follow-up periods (eg, 30 minutes, 1 hour, or longer) are warranted to assess the duration and stability of the responses.

### Future Directions and Conclusions

Although gentle eye massage can offer therapeutic benefits, excessive or improper pressure may lead to corneal damage, intraocular structural issues, soft tissue trauma, and vascular complications. Patients with preexisting ocular conditions (eg, glaucoma, keratoconus, or recent eye surgery) should exercise caution and seek medical advice before eye massage. More research is needed to fully understand the long-term implications of repeated ocular massage on eye health, including the durability of IOP reduction, cumulative tissue effects, and optimal massage parameters. Future clinical trials involving diverse patient populations and extended follow-up periods will be essential to determine the safety and efficacy of ocular massage as a nonpharmacological therapeutic strategy.

## Data Availability

The datasets generated and analyzed during this study will be available from the corresponding author on reasonable request.

## Appendix

Multimedia Appendix 1 Modified Quality of Vision Questionnaire.

## Abbreviations

AE: adverse event
AS-OCT: anterior segment optical coherence tomography
IOP: intraocular pressure
PI: principal investigator
SC: Schlemm canal
SPIRIT: Standard Protocol Items: Recommendations for Interventional Trials
TM: trabecular meshwork
TMUEH: Tianjin Medical University Eye Hospital
