# Supplementary material for: Impact of Ocular Massage on Intraocular Pressure and Schlemm Canal Dimensions in Healthy Adults: Protocol for a Randomized Controlled Trial
Source: JMIR Res Protoc. 2026 Feb 26;15:e78864. doi: 10.2196/78864 (PMC12982946; doi:10.2196/78864)
Supplement: Multimedia Appendix 1 [file resprot_v15i1e78864_app1.docx]

**Multimedia Appendix 1.** Modified quality of vision questionnaire.

| **Items** | **Symptom** | **Question** | **Severity score** | | | |
| --- | --- | --- | --- | --- | --- | --- |
|  |  |  | **0** | **1** | **2** | **3** |
| 1 | Glare | How often do you experience glare? | Never | Occasionally | Quite often | Very often |
|  |  | How severe is the glare? | Not at all | Mild | Moderate | Severe |
|  |  | How bothersome is the glare? | No at all | A little | Quite often | Very |
| 2 | Halo | How often do you experience haloes? | Never | Occasionally | Quite often | Very often |
|  |  | How severe are the haloes? | Not at all | Mild | Moderate | Severe |
|  |  | How bothersome are the haloes? | No at all | A little | Quite often | Very |
| 3 | Starburst | How often do you experience starbursts? | Never | Occasionally | Quite often | Very often |
|  |  | How severe are the starbursts? | Not at all | Mild | Moderate | Severe |
|  |  | How bothersome are the starbursts? | No at all | A little | Quite often | Very |
| 4 | Hazy Vision | How often do you experience hazy vision? | Never | Occasionally | Quite often | Very often |
|  |  | How severe is the hazy vision? | Not at all | Mild | Moderate | Severe |
|  |  | How bothersome is the hazy vision? | No at all | A little | Quite often | Very |
| 5 | Blurry Vision | How often do you experience blurred vision? | Never | Occasionally | Quite often | Very often |
|  |  | How severe is the blurred vision? | Not at all | Mild | Moderate | Severe |
|  |  | How bothersome is the blurred vision? | No at all | A little | Quite often | Very |
| 6 | Distorted Vision | How often do you experience distortion? | Never | Occasionally | Quite often | Very often |
|  |  | How severe is the distortion? | Not at all | Mild | Moderate | Severe |
|  |  | How bothersome is the distortion? | No at all | A little | Quite often | Very |
| 7 | Double or multiple images | How often do you experience double or multiple images? | Never | Occasionally | Quite often | Very often |
|  |  | How severe are the double or multiple images? | Not at all | Mild | Moderate | Severe |
|  |  | How bothersome are the double or multiple images? | No at all | A little | Quite often | Very |
| 8 | Visual Fluctuation | How often do you experience a fluctuation in your vision? | Never | Occasionally | Quite often | Very often |
|  |  | How severe is the fluctuation in your vision? | Not at all | Mild | Moderate | Severe |
|  |  | How bothersome is the fluctuation in your vision? | No at all | A little | Quite often | Very |
| 9 | Focus Difficulty | How often do you experience focusing difficulties? | Never | Occasionally | Quite often | Very often |
|  |  | How severe ate the focusing difficulties? | Not at all | Mild | Moderate | Severe |
|  |  | How bothersome are the focusing difficulties? | No at all | A little | Quite often | Very |
| 10 | Depth perception | How often do you experience difficulty judging distance or depth perception? | Never | Occasionally | Quite often | Very often |
|  |  | How severe is the difficulty judging distance or depth perception? | Not at all | Mild | Moderate | Severe |
|  |  | How bothersome is the difficulty judging distance or depth perception? | No at all | A little | Quite often | Very |
| 11 | Overall quality of vision | 0 = Extremely Poor, 10 = Excellent | 0, 1, 2, 3, 4, 5, 6, 7, 8, 9, 10 | | | |
| 12 | Ocular pain | 0 = No Pain, 10 = Extremely painful | 0, 1, 2, 3, 4, 5, 6, 7, 8, 9, 10 | | | |
| 13 | Ocular discomfort | 0 = No Discomfort, 10 = Extreme Discomfort | 0, 1, 2, 3, 4, 5, 6, 7, 8, 9, 10 | | | |
